# Book Reviews

**Published:** 1816-01

**Authors:** 


### CRITICAL ANALYSIS
### OF RECENT PUBLICATIONS
IN THE
### DIFFERENT BRANCHES OF PHYSIC, SURGERY, AND MEDICAL PHILOSOPHY.

*Physiological Researches on Life and Death;* by XAVIER BICHAT. Translated from the French, by F. GOLD, Member of the Royal College of Surgeons, London. 8vo. pp. 283. Bristol; printed for Longman and Co. London.

A LIFE of Bichat is prefixed to this work, the more interesting particulars of which are already before our readers. Researches on life and death can scarcely be productive of any practical advantages excepting in one important inquiry, namely, the signs of death, and the best means of resuscitation after an apparent suspension of the actions of life. Whatever is said must lead to such inquiries, and indeed the phenomena of both are intimately intermixed. We should therefore have been better pleased had the title contained a reference to that part of medicine, as it would have been the means of leading to some object, through a series of experiments, most of which have been anticipated by Mr. Hunter with much more satisfactory inferences. We shall, however, pursue our analytic process, as the only impartial mode of treating an author.

First, then, for the definition of life. "Life, it is said, consists in the sum of the functions, by which death is resisted." This is not expressed so well as by our English physiologist, who calls life the power of self-preservation from the changes which take place in dead animal matter. In the next position both agree. "The principle of life is unknown in its nature, and can only be appreciated by its phenomena." To what purpose then are researches after what can never be discovered, and why is not the title of the work An Inquiry into the Phenomena which distinguish living from dead Animal Matter, and into the Means by which Life is preserved or destroyed in the various parts or in the whole of an Animal. Such a title would have kept our minds awake to some practical object, to the avoiding of every means of destruction, or the providing as much as possible against their effects. A remark follows that there is a superabundance of life in the child; that is, that, besides the usual powers of support, there are powers of increase, and that the powers of restoration are greater.

After these preliminaries, the subject opens with "the division of life into animal and organic life." This comprehends a distinction of those actions by which life is supported, which vegetables possess as well as animals, and those properties which are peculiar to animals, by which they are connected with external objects, by which they feel, reflect, and move, according to the influence of certain impressions, and are able to communicate, in some cases, by their voice, their desires, fears, pleasures, and pains.

The aggregate of the functions of the first order, M. Bichat names *organic* life, because all organised beings, whether animal or vegetable, enjoy it more or less, because *organic texture is the sole condition necessary to its existence.* The sum of the condition of the second class, because it is exclusively the property of the animal, he names animal life.

We cannot help arresting the attention of our readers for a moment, to show the superior accuracy of our English physiologist. If life, said he, depends entirely on the construction of certain parts (or texture, as M. Bichat calls it), we should find those parts different in the living and dead subject; but this is not the case. "Life, therefore, is a something superadded, the nature of which we neither do nor can ever comprehend." It must be admitted that M. Bichat admits our incapacity to comprehend the nature of life. Why then, we would ask, does he so far forget himself as to say that "organic texture is the only condition necessary to its existence." If by this he would have us understand that this organic texture is all we can discover in organic life, we might ask, what more can he discover in animal life? We see certain organs, by which we perceive that animals live, and are supported. We see the same organs when the actions of life are interrupted. In animals we see organs for similar purposes, and others by which the animal is enabled to perceive external objects, and communicate those sensations which such objects excite. These organs we can not only discover, but even imitate their structure; but we can never discover that power by which impressions are received or communicated. Life, therefore, (we repeat it in the words of our countryman) is a property we do not understand, and of which we can only trace the phenomena by which it is distinguished.

We transcribe the following section as a specimen of the near approach to metaphysics which every author must make when he attempts to state analytically the various properties of living bodies.

"§ II. *Subdivision of each of the two lives into two orders of functions.*—The animal and the organic life are each of them composed posed of two orders of functions, which succeed each other, and are concatenated in an inverse direction.

"In the animal life, the first order is established from the exterior of the body, towards the brain; the second from the brain towards the organs of locomotion and the voice. The impression of objects successively affects the senses, the nerves, and the brain. The first receive, the second transmit, the third perceives the impression. The impression in such way received, transmitted, and perceived, constitutes sensation.

"The animal, in the first order of these functions, is almost passive; in the second, he becomes active. This second order is the result of the successive actions of the brain (where volition has been produced in consequence of the previous sensation); of the nerves, which transmit such volition; and of the locomotive organs and voice, which are the agents of volition. External bodies act upon the animal by means of the first order of these functions; the animal re-acts upon them by means of the second.

"In general there exists between the two orders a rigorous proportion: where the one is very marked, the other is put forth with energy. In the series of living beings, the animal which feels the most moves also the most. The age of lively perception is that also of vivacity of motion; in sleep, where the first order is suspended, the second ceases, or is exercised only with irregularity. The blind man, who is but half alive to what surrounds him, moves also with a tardiness which would very soon be lost, were his exterior communications to be enlarged.

"A double movement is also exercised in the organic life; the one composes, the other decomposes, the animal. Such is the mode of existence in the living body, that what it was at one time it ceases to be another. Its organization remains unaltered, but its elements vary every moment. The molecules of its nutrition, by turns absorbed and rejected, from the animal pass to the plant, from the plant to inorganic matter, return to the animal, and so proceed in an endless revolution.

"To such revolution the organic life is well adapted. One order of its functions assimilates to the animal the substances which are destined to nourish him, another deprives him of these substances, when, after having for some time made a part of it, they are become heterogeneous to his organization.

"The first, which is that of assimilation, results from the functions of digestion, circulation, respiration, and nutrition. Every particle which is foreign to the body before it becomes an element of it, is subject to the influence of these four functions.

"When it has afterwards concurred for some time to the formation of the organs, the absorbents seize on it, and throw it out into the circulatory torrent, where it is carried on anew, and from whence it issues by the pulmonary or cutaneous exhalations, or by the different secretions by which the fluids are ejected from the body.

"The second order, then, of the functions of the organic life, or that of decomposition, is formed of those of absorption, circulation, exhalation, and secretion.

"The sanguiferous system, in consequence, is a middle system, the center of the organic life, as the brain is the center of the animal life. In this system, the particles which are about to be assimilated are circulated and intermixed with those which, having been already assimilated, are destined to be rejected; so that the blood itself is a fluid composed of two parts; the one, the pabulum of all the parts of the body, and derived from the aliment; the other, excrementitious, composed of the wrecks and residue of the organs, and the source of the exterior secretions and exhalations. Nevertheless these latter functions serve also, at times, the purpose of transmitting without the body the products of digestion, although such products may not have concurred to the nourishment of the parts. This circumstance may be observed when urine and sweat are secreted after copious drinking, the skin and the kidneys being at such times the excreting organs, not of the matter of the nutritive, but of that of the digestive process; the same also may be said of the milk of animals, for this is a fluid which certainly has never been assimilated.

"There does not exist between the two orders of the functions of the organic life the same relation which takes place between those of the animal life. The weakness of the first by no means renders absolutely necessary a decrease of action in the second. Hence proceed marasmus and leanness, states in which the assimilating process ceases in part, the process of excretion remaining unaltered.

"Let us leave, then, to other sciences, all artificial method, but follow the concatenation of the phenomena of life, for connecting the ideas which we form of them; and we shall perceive, that the greater part of the present physiological divisions afford us but uncertain bases for the support of any thing like a solid edifice of science.

"These divisions I shall not recapitulate; the best method of demonstrating their inutility will be, if I mistake not, to prove the solidity of the division which I have adopted. We shall now examine the great differences which separate the animal existing without from the animal existing within, and wearing itself away in a continual vicissitude of assimilation and excretion."

Those who are fond of pompous nothings, or, as is said of some gothic buildings, "passages which lead to nothing, and windows which exclude the light," may study the above passage, and, when they have made themselves masters of it, will find themselves exactly where they were before. Such reasoning as this is too plain to require more than to remind us of it; and, unless we get it by heart, we shall find it necessary to recapitulate as much as we have occasion for, whenever any inference is to be drawn from it. It therefore tends only only to disgust the modest inquirer, who can hardly believe he has been studying what is already familiar to him, or it elevates the already-confident too much in his own opinion, when he conceives that he has been studying a profound proposition, of which he has made himself master with so much facility.

The second chapter is on the general difference of the two lives, with regard to the outward form of their respective organs. There is much ingenuity and interest in these sections. The symmetry of the parts connected with animal life is shown, not only in most of the organs of sense being double, and exactly corresponding with each other, but also in the regularity of their conformation in most subjects, and the inconvenience which arise from any deficiency, either in symmetry or uniformity. On the contrary, it is very justly remarked, that in the organic life all the parts subservient to those purposes are irregularly disposed; that there is nothing to correspond in figure with the heart, the liver, or spleen; that, though there are two kidnies, they are rarely exactly similar, and the lobes of the lungs always different; that not only does this want of symmetry prevail, but also a want of uniformity in different subjects; that the heart itself varies in size, and the disposition of the intestines also; that the livers of different subjects are seldom exactly alike, and the uncertain size of the uterus is universally known and remarked. The blood-vessels vary considerably, and the glandular system still more.

The third chapter begins with showing the general difference of the two lives, with regard to the mode of action of their respective organs. In the first or animal life, it is not difficult to prove that any irregularity in the configuration of the organs of sense must produce a want of harmony in their effects on the sensorium. If the eyes are different in figure, the axis of vision is different, and, in order to view an object distinctly, one of them must be shut. If artificial assistance is used for one, the other is usually shut. The ears, we are told, must hear alike, or the consequence will be a confused sense of hearing, inconsistent with the power of discriminating harmony from discord. This is a question we leave to be decided by musicians. The subject is considered as relating to the other senses, in which it is shown, that to produce a complete harmony in their whole effect, there must be a complete correspondence between the organ and the sensorium, and even the various sympathies connected with them. We extract the following passage to show that in some of his opinions Spurzheim is supported by this very popular writer.

"The

"The external senses are the natural excitants of the brain. The functions of the brain succeed to theirs; and this organ would but languish, were it not to find in them the principle of its activity. From sensation follow perception, memory, and imagination; from these the judgment. Now it is easy to prove, that these different functions, commonly known by the name of the internal senses, are governed in their actions by the same laws which influence the external senses; and that, like them, they approach the nearer to perfection in proportion to the degree of harmony existing in the symmetrical parts in which they have their seat.

"Let us suppose, for instance, one hemisphere of the brain to be better organised, and therefore susceptible of livelier affections, than its fellow: in such case, the perception of the individual would be confused, for the brain is to the soul what the senses are to the brain; it transmits to the soul the impressions conveyed to it by the senses, as the senses convey to the brain the impressions made upon them by external objects. But, if the defect of harmony in the external senses confuse the perception of the brain, why may not the soul perceive but confusedly, when the two hemispheres of the brain are unequal in power, and incapable of blending into one the double impression which is made upon them?

"The memory is the faculty of re-producing former sensations; the imagination that of creating new ones: now, in the act of remembering or imagining, each hemisphere of the brain appears to re-produce, or to create a sensation of its own. If both do not act alike, the perception of the mind, which ought to be the result of the two sensations united, will be inexact and irregular. But it is evident that there will be a disparity in the two sensations, if there be a disparity in the two halves of the brain in which they have arisen, and since the general foundations of the judgment are made up of the faculties of perception, memory, and imagination, if these be confused, the judgment itself must be confused also.

"We have now supposed an inequality of action in the hemispheres of the brain, and inferred, that the functions would in this supposition be imperfect; but what as yet is only supposition, in a variety of instances can be proved to be a fact; for nothing is more common than to find, in consequence of compression on either hemisphere by blood, pus, or exostosis, a variety of alterations in the intellectual functions.

"Even when all appearances of actual compression have vanished, if in consequence of that which has been experienced, a part of the brain remain enfeebled, the same alterations of mental power will be found to be prolonged. If both hemispheres of the brain, however, be affected equally, the judgment, though weaker, will be more exact. Perhaps it is thus that we should explain those observations so frequently repeated, of an accidental stroke upon one side of the head having restored the intellectual functions, which had long remained dormant in consequence of a blow received upon the other side.

"I now

"I now conceive myself to have proved, that with inequality of action in the hemispheres, there must be confusion of intellect. I have also pointed out some states of disease, in which such confusion is evidently the effect of inequality of action so occasioned: here we see the effect and its cause; but may we not, from analogy, infer a similar cause where we see a like effect? when the judgment is habitually incorrect, and all the ideas wanting in precision, may we not be induced to believe, that there does exist a defect of harmony in the action of the two hemispheres of the brain? We see inaccurately if nature have not given to both eyes an equal power; we perceive and judge inaccurately, in like manner, if the two sides of the brain are naturally dissimilar. The most correct mind, and the soundest judgment, pre-suppose in the hemispheres a perfect harmony of action; and what a multiplicity of shades do we not behold in the operations of the understanding? It is probable that they all of them correspond to so many varieties in the proportions of power in the hemispheres. Could we squint with the brain as we do with the eyes—that is to say, could we receive impressions on one hemisphere only, and form from thence our determinations, we might then command at will a precision in our intellectual operations; but such a power does not exist."

"I wish to observe (concludes our author) in finishing this section, that in pointing out the different derangements which take place in the animal life, from the want of harmony in the organs, I have only pretended to assign a single isolated cause of such derangements; I am well aware that a thousand other causes besides dissimilarity in the hemispheres of the brain may affect the operations of the mind."

The opinion of a discordance of action arising from any difference in the organs is carried even to locomotion. Bichat does not consider, like Spurzheim, that the right side is originally stronger than the left, but that the difference arises from habit and education; and that for want of similar powers in both hands and both sides of the body, there is a deficiency in the harmony of action of the whole, which can only be remedied by giving less power to the right and more to the left, so as to reduce both to a level. This he conceives absolutely necessary to graceful dancing, and other feats of agility. Necessary as he conceives harmony to all the organs of animal life, M. Bichat proceeds to show that in organic life it is of no consequence.

"What detriment (he asks) would it be to the general health of the individual, should one of his kidneys be stronger than the other, and secrete more urine; should one of his lungs be better unfolded than the other, admit more venous, and send out more arterial, blood; should a less organic force be the lot of the salivary glands on one side than on the other side of his body? The simple function, to which both organs concur, would not be performed formed less perfectly. Whenever but a slight fulness supervenes on one side of the liver, spleen, or pancreas, the sound part makes up for the defect, and the function is little disturbed. The circulation also remains unaltered among the frequent variations in the vascular system on each side of the body, whether such variations exist naturally, or whether they arise from some artificial obliteration of the larger vessels, as in aneurism."

Hence he remarks, with much judgment, that the little varieties in the formation, and even the actions, by which mere organic life is supported, viz. a quicker or slower circulation, a greater or less quantity of bile, or urine, or fæces, are of only temporary existence, and altogether unimportant to the general economy.

The fourth chapter is on the difference of the two lives with respect to duration [continuance] of action. Respecting organic life, it is shown that the shortest suspension endangers, and that continuance of such suspension extinguishes, life. All the secretions, it is remarked, are continually going on, though in various degrees, and with a mutual dependance on each other, as the circulation, respiration, &c. On the contrary, in the *animal life intermissions are absolutely necessary, not merely remissions. All the senses are fatigued by long exertion, and lose their sensibility, till they are relieved by rest. The brain must, after intense action, have rest; the muscles also. Mr. Hunter, with his usual accuracy, remarked, that even the muscles of involuntary motion, as the heart, which Bichat considers as forming a part of organic life, require rest. The action of the heart, he remarked, was alternated with rest in every systole and diastole.

A section follows on sleep, as an illustration, according to its degree, of the remission or intermission of animal life.

Another distinction in the character of the two lives consists in the independance of one, and dependance of the other, on habit. Animal life, it is shown, is almost governed by habit, as we say, commonly, that habit is second nature. The effect, however, of habit is shown to blunt sentiment or feeling, but at the same time to improve judgment. The latter position is unquestionable. The former is liable to many exceptions. A fine country is said to please at first sight, but to satiate by constant view. This often depends on other associations. The taste, so far from being blunted, is often more alive to particular impressions from certain

---

* Among the innumerable errors of the press, this is not the least: instead of contrasting the two lives, the translator has termed them both organic. See Translation, p. 48.

food food or liquors, in proportion as it is more accustomed to them. This M. Bichat would call judgment; but, if the impression is lively, it cannot be said to be blunted. In many instances the aphorism is very just. We may be reconciled to many things besides the introduction of a bougie, by frequent habit. If, by this remark, the author only refers to the necessity of some intermission in the exercise of the organs, he means no more than was contained in his former chapter. That habit improves judgment, cannot be questioned.

In the organic life, on the contrary, it is shown that habit has no influence, unless we refer to some actions which are partly voluntary, such as the evacuation of excrement, the habit of the stomach for certain kinds of food and at certain periods. These, therefore, are considered as holding a middle place between the two lives, or as partaking of both.

The sixth chapter is on the difference of the two lives with respect to mental affections. The introduction is so well expressed that we shall transcribe it at length.

"It is necessary to consider, under two relations, those acts which, little connected with the material organization of animals, are derived from this principle, so little known in its nature, but so remarkable as to its effects, the center of all their voluntary motions; and on the subject of which there would have been less dispute, if philosophers, instead of attempting to reach its essence, had been contented with analyzing its operations. These actions, which we shall consider more especially in man, with whom they are the most perfect, are either purely intellectual, and relative to the understanding only, or they are the immediate product of the passions. Examined under the first point of view, they are the exclusive attribute of the animal; under the second, of the organic life."

The first section, that "whatever relates to the understanding belongs to animal life," contains nothing which can admit of a question. The second, that "whatever relates to the passions belongs to organic life," requires some illustration. It is a very important passage, containing many very valuable and ingenious remarks. To enter fully into it, the passage must be carefully perused: we shall only offer the outlines.

That the passions depend on organic life, the author endeavours to prove by showing that the organs will excite all the passions, and that all the organs will be affected each by its appropriate passion. Anger affects the heart, and the whole circulating system. Terror affects the same parts, but in a different ratio. Sadness and sorrow are nearly analogous, and only less in degree. Respiration, digestion, and many of the secretions, are influenced by the passions; and these secretions influence the passions. The very language of the vulgar, observes our author, would direct us right in these respects, notwithstanding the influence of philosophy. Our common expressions, *une tête forte*, *bien organizée*, a strong or well organized head,* in speaking of a sound understanding; *un bon cœur*, a heart, sensible to indicate feelings, or the prevalence of amiable passions. The very expressions of fury thrilling through the veins, and rousing the gall; of joy making the heart leap; of jealousy distilling poison into the heart; are, we are assured, by no means poetical expressions, but real descriptions of nature. At length the author goes a step further; and, if we are not ready to follow him throughout, we are ready to admit that we are still less disposed to contradict him. He ventures to assert that anger and love inoculate, if we may so express it, into the humours, particularly the saliva, a radical vice, to which he imputes the danger of the bite of animals agitated by such passions. The passions of the nurse, he conceives, impress on the milk an injurious taint, from whence various diseases affect the infant. This is tender ground to tread upon. We shall therefore only refer our readers to the remarks we ventured on Mr. Parkinson's case, in our review of Dr. Parry's opinions concerning Hydrophobia. The effect on infants is well known; but may be readily accounted for by the want of health in a nurse under the prevalence of any unfavourable passion, as it is not pretended that the child's temper becomes similar to the nurse's.

"The ancients (concludes our author) were better acquainted with the laws of the economy than our modern mechanicians, in supposing that our bad affections were evacuated by purgatives, together with the noxious humours of the body. By disembarrassing the primæ viæ, they got rid of these affections. In fact, how dark a tint does the fulness of the gastric viscera cast upon the countenance! the errors of the first physicians on the subject of the atrabilis, were a proof of the precision of their observations on the connexion of these organs with the state of the mind.

"In this way every thing tends to prove, that the organic life is the term in which the passions end, and the centre from whence they originate. But we shall be asked, perhaps, why vegetables, which live organically, do not offer any vestige of them? The reason seems to be, that besides their want of the natural excitants

* Our English expression is a *cool head*, with which Voltaire was so much pleased, that he adopted and translated it *une tête froide*, in describing the character of Marlborough. See Mem. of Louis XIV.—Edit.

of of the passions, namely, the external apparatus of the senses, they are wanting also in those internal organs which concur most especially to their production, such as the digestive system, that of the general circulation, and that of the great secretions, which are remarked in animals.

"Such are the reasons also why the passions are so obscure in the Zoophytes, in worms, &c.; and why, in proportion as the organic life becomes more simple in the series of animals, and loses its important viscera, the passions are less observable."

It is next remarked, that "the passions modify the actions of the animal life, though seated in the organic life." The meaning of this is, that, though the seat of the passions, as was just observed, is in the viscera, or parts subservient to the mere existence and preservation of life, yet they often affect those parts which are usually subservient only to volition and reason. This is illustrated by the following case. A person, whilst in company, learns an event which he wishes to conceal. His countenance instantly changes, his brows are contracted, and his features moulded according to the nature of the passion. These are considered as sympathetic phenomena, arising from certain abdominal viscera suddenly affected by this passion, and which consequently appertain to organic life. When the man so far recovers himself as to be enabled voluntarily to exert the muscles of his countenance, the above effects cease, but the internal feeling is the same. The action of the brain has here surmounted that of the lower viscera, and animal life has resumed its proper influence and power over the voluntary muscles.

This chapter concludes with a section "on the epigastric centre, to show that it does not exist as some authors have assumed." That the brain is the source of all voluntary motion, no one can question; but whoever has attempted to ascertain the seat of the passions, has generally ended as he began. This M. Bichat very justly ascribes to the variety of seats, as well as their uncertainty. In some men, parts are affected by the same passions as affect others in different viscera. Add to this, such is the great uncertainty of the impression from the affections of different abdominal or thoracic viscera, that the pain is no certain index of the seat or extent of the mischief. This chapter contains a long note on the sympathetic nerve, which the translator has passed over. Perhaps, had he omitted much more, the work might not have been less valuable, but it is certainly less faithful, as a translation. Even the passage to which the note refers is unfaithfully translated. These errors are unimportant; but it is our duty to notice them.

 The

The conclusion is, that though for the sensations there exists one common centre in the brain, yet for the passions there is no certain seat; and that this confusion arises, in part, from the propinquity of all the vital viscera contained in the abdomen and thorax. The following illustrations are attached, to show the variety of sentiments dependant on the form of the cranium, and the probable existence of something of the kind in the lower viscera, on which the force and nature of the passions depend.

"In determining the facial angle, Camper has thrown much light upon the proportion of intelligence enjoyed by the several classes of animals. It appears that not only the functions of the brain, but that all those of the animal life which are centered there, have this angle for the measure of their perfection.

"It would be a very pleasing thing could we indicate in the same way a measure, which, assumed from the organs of the internal life, might fix the rank of each species with regard to the passions. The dog is much more susceptible than other animals of the sentiments of gratitude, of joy, of sorrow, of hatred, and of friendship: has he any thing more perfect in his organic life? The monkey astonishes us by his industry, his disposition to imitate, and by his intelligence; his animal life is certainly superior to that of every other species. Other animals, such as the elephant, interest us by their attachment, their affections, their passions; they delight us also with their address, and the extent of their intelligence. With them the cerebral centre and the organic viscera are perfect alike.

"A rapid glance over the series of animals will shew us also, that in some of them the phenomena which arise from sensation predominate over those which have their origin in the passions; in others we shall see the latter superior in power to the former, and in others, again, a balance established between the two. These circumstances, which we remark in the long chain of animated beings, we may remark in the human species alone. In one man, the passions are the great principle of motion; the influence of his animal life is constantly surpassed by that of his organic life, and incessantly induces him to act in a way to which the will is almost a stranger, and which often entails upon him the bitterest regret, when his animal life resumes its empire. In another man, the animal life is the stronger of the two. In such case, the understanding seems to be augmented at the expence of the passions, the latter remaining in that silence to which the organization of the individual has condemned them.

"That man enjoys the happiest constitution in whom the two lives are balanced, in whom the cerebral and epigastric centers exercise the one upon the other an equal action, whose intellect is warmed, exalted, and animated by the passions, but whose judgment makes him at all times master of their influence.

"It is this influence of the passions over the actions of the animal animal life, which composes what is named the character. Character, as well as temperament, depends upon the organic life, possesses all its attributes, and is a stranger to the will in all its emanations; for our exterior actions form a picture of which the ground and design do indeed belong to the animal life, but upon which the organic life extends the shading and colouring of the passions. The character of the individual is constituted by such shades and colours.

"The alternate predominance of the two lives has been remarked by almost all philosophers. Plato, Marcus Aurelius, Bacon, St. Augustine, St. Paul, Leibnitz, Van Helmont, Buffon, and many others, have recognized in man two principles, by one of which we become the masters of all our moral actions, by the other the contrary. We have nothing to do with the nature of these principles. Our business is with their phenomena; we shall analyze the relations by which they are united."

The seventh chapter, on the "general differences of the two lives with respect to vital power," commences with an introductory section, which the author calls a digression, and apologises for its length. We could almost wish it had been longer, and yet we are fearful it would be to little account. Its object is to show that the study of animal life should always be distinct from what are called the accessory sciences. That even animal chemistry is fallacious, inasmuch as all our secretions differ at different periods, and under different circumstances. A lively illustration is given of the consequences which follow from the application of the language of one science to another. Had physiology been cultivated before the other branches of natural history, the author is persuaded that the language of the former would have been applied often to the latter. Rivers would have flowed from the tonic action of their banks; crystals united from mutual excitement; and planets have moved by irritating each other at their vast distances. Unreasonable as this may now appear, it is scarcely less absurd than the application of mechanical or chemical language to the operations or phenomena of living bodies.

The remarks on "the difference between the vital properties and those of texture," should, in the description of the latter, have been confined to elasticity, the only property common to the living and dead parts of a body.

The "two kinds of sensibility, viz. the animal and organic sensibility," refer principally to the difference between the sensibility of a part to certain impressions, and the communication of such impression to the mind or animal life. Here the author takes occasion to remark, that every part is capable of the latter under certain circumstances. Though periosteum, periosteum, bone, and tendon, have been supposed insensible, yet this is only in degree. Under inflammation they are as sensible as other parts.

We would willingly follow our author through the remaining sections of this chapter, which contain many useful remarks, mixed with adages so simple, as to appear almost unnecessary in a philosophical work. In considering "the relation which exists between the sensibility of each organ to foreign bodies," the author gives a *slight* view of the theory of inflammation, slight indeed when we consider the importance of the subject, and the manner in which our English physiologist has traced all its leading steps.

In the chapter "on the development of organic life," M. Bichat enters into the question of moral sentiment, as connected with the passions; and, conceiving them the effect of an organization which cannot be altered, concludes that all we can expect from education is to moderate those which are bad, and improve the good.

The last chapter of this part of the work is on the termination of the two lives. Here we have a gloomy picture of old age, but interesting, inasmuch as it prepares us for the loss of life, or renders us almost insensible of the change. The animal life having ceased before the organic, reduces man almost to the condition of a vegetable. In all this the author confines himself to death from old age: the various other modes of dying are to be considered in the second part of this work. There is, however, a section at the close, to show that "organic life in natural death does not terminate as it does in accidental death." This we shall transcribe, as it leads to a few remarks which we have often felt impatient to make.

"The organic life remains with the old man after the almost total loss of his animal life, and terminates in a very different manner from that which is exemplified in the case of violent and sudden death. The latter has two periods, the first of which is marked by the sudden cessation of respiration and the circulation; the second by the slow and gradual extinction of the other organic functions.

"The parietes of the stomach, for instance, continue to act upon the aliment which may be found there; the juices of the stomach continue to dissolve it. The experiments of the English and Italian physicians upon absorption (experiments the whole of which I have repeated) have proved that this function not unfrequently remains in a state of activity after the general death of the body, and, if not as long as some have supposed, at least for a very considerable interval. Discharges of urine and fæces are often observed to take place many hours after sudden death.

"The process of nutrition also continues to be manifest in the hair hair and in the nails: the same would doubtless be the case in all the other parts, as well as in the secretions, could we observe the insensible movements of which their functions are the result. The heart of the frog being taken away, the capillary circulation may still be seen under the influence of the tonic powers. The body is very slow also in losing its animal heat.

"I might augment the above observations with a number of others, which would go to prove the same assertions; on the contrary, in the death which is the effect of old age, the whole of the functions cease, because they have each of them been successively extinguished. The vital powers abandon each organ by degrees, digestion languishes, the secretions and the absorptions are finished, the capillary secretions become embarrassed; lastly, the general circulation is suppressed. The heart is the ultimum moriens.

"Such, then, is the great difference which distinguishes the death of the old man, from that which is the effect of a sudden blow. In the one, the powers of life begin to be extinguished in all the parts, and cease at the heart; the body dies from the circumference towards the centre. In the other, life becomes extinct at the heart, and afterwards in the parts. The phenomena of death are seen extending themselves from the centre to the circumference."

By this passage it is evident that Bichat refers to premature rather than to sudden death, and takes death by a violent blow in much too general a sense. Death from a violent blow on the stomach is frequently so instantaneous in every part, that we can trace no appearance or property of life either near the centre or circumference. It is when this effect is instantly produced on animals in previous high health, that Mr. Hunter and others have discovered the digestion of the stomach itself. It is curious to see how gently the French physiologist touches on this subject; and, without mentioning a name, or even hinting the most important part of the experiment, just refers us to the English and Italian physicians. On this subject we shall have more to say in our next Number, when we shall offer our analysis of the second part of this work.

---

*An Inquiry into the Causes of the Motion of the Blood; with an Appendix, in which the Process of Respiration and its Connexion with the Circulation of the Blood are attempted to be elucidated.* By JAMES CARSON, M.D. Physician to the Workhouse, the Fever Hospital, and to the Asylum for the Pauper Lunatics at Liverpool; and in Charge of the Military Hospital at that place. 8vo. pp. 250. Liverpool; printed for Longman and Co.

IT is certain that in the whole study of natural history, nothing can be considered uninteresting or unimportant.

 Yet

Yet it is the duty of every practical man, and such we must consider every physician, to recollect the first adage of our immortal master, 'ο βιος βραχυς. To read many such books as the one before us, and to study so as to become master of their full meaning, would consume more of the useful portion of life than any physician has to spare. It may be asked, How much more must it cost the writer? This we pretend not to answer. It is enough for us to complain of the great difficulty we felt in keeping up our attention, whilst we were attempting to analyse the work in such a manner as to interest the reader. But all our endeavours were fruitless. We do not complain of a book whose meaning is not obvious at first sight; on the contrary, we are ready to admit that few good things, if, at the same time, new, can be placed in so perspicuous a form as not to require a second, and, in some parts, a third perusal. But then we are always enticed to persevere by the practical advantages we see gradually opening before us. Without this we lose the great sweetener of labour,—the hopes of reward; and it is impossible to preserve our patience, even if we retain our temper.

We confess our disappointment was the greater in the present case, because, from the character of Dr. Carson, we had raised our expectation the higher. We expected at least a series of experiments, and fondly flattered ourselves that some light might have been afforded on the hitherto inexplicable subject of the passage of the blood from the arteries to the veins. Dr. Carson, indeed, suggests some advantages from his inquiry relative to the doctrine of inflammation; but we have in vain endeavoured to discover them. If we understand him, he conceives the circulation of the blood may be reduced to the laws of hydrostatics, and for this purpose that we need only attend to the resilient state of the lungs at every expiration, the alternate contraction and dilatation of the heart in each systole and diastole, and also to its confinement in the pericardium. We cannot suppose this is all that the book contains; and, in order to do justice to the author, we very eagerly sought for some detached part, which might be fairly extracted, as a specimen of his intentions, and the mode in which he has executed them.

The appendix seems to furnish the best chance of extracts least complicated with other matter. It commences with a general view of the process of respiration, and its connection with the circulation. As this subject is pretty generally understood, the reader will perceive the slight objections made by the author to the commonly-received doctrine; and, if he cannot cannot exactly ascertain his whole meaning, he may at least learn his mode of expressing it.

"In this explanation we discern a cause, interwoven with the structure of the lungs, rendered permanent and certainly efficacious, by the position and mechanism of the chest and its contents, clearly explanatory of all the phenomena, and, at all times, adequate to the accomplishment of that elevation which the diaphragm requires for the performance of its important functions.

"When the diaphragm has been distended upwards, by the continued operation of the balance thrown, in the manner that has been explained, against its inferior surface to such an extent that the muscular fibres of which it is chiefly composed have been stretched beyond their natural length, these fibres, in consequence of the stimulus of pain, of volition to which this muscle is in a certain degree subservient, or of some other cause, exert a contractile effort, which, being more powerful than its antagonist, restores the diaphragm from the conical to the flat condition.

"No sooner has the energy of the muscular action, agreeably to the nature of that action, been relaxed to a certain extent, than the antagonist but weaker power becomes in its turn predominant, and, by the constancy of its operation, restores the cone formed by the diaphragm to those dimensions from which it had been reduced, and, on its restoration to which, the dormant contractile power is again roused.

"The contractile power of the diaphragm, in conformity with the laws of muscular motion, is irregular, remitting, and sometimes altogether quiescent. The elasticity of the lungs, on the other hand, is equal and constant. The superior energy of the former is balanced by the permanency of the latter. By the advantage which the inferior power, from the uniformity of its operations, is enabled to take of the remissions of its more powerful antagonist, the ground which had been lost is recovered, and the contest prolonged; that contest in which victory declaring on one side or the other is the instant death of the fabric.

"If the question were asked, What constitutes the main spring of life? there could, I think, be no hesitation in replying that it consists in the contest which is maintained between the elasticity of the lungs and the irritability of the diaphragm, supposing the heart, as it certainly must be considered, an appendage of the latter."

Towards the close, the author attends to a difficulty in one part of his theory which, perhaps, has already occurred to the reader.

"The lungs of the unborn fœtus, as they are in a state of complete collapse, can have no resiliency, and, therefore, no influence in producing the expansion of the chambers of the heart after contraction, in the manner supposed in the adult. But the heart beats and the blood circulates in the fœtus in utero as well as after after birth. Here then is a case in which the heart must be dilated after contraction, without the assistance of that cause to which this effect has been chiefly ascribed in the preceding inquiry; and, if the effect can be produced without the supposed cause in one instance, it may be urged that it is unnecessary and unreasonable to have recourse to it in any other.

"With a view to the explanation of the manner in which the blood is circulated in the fœtus, and to the removal of the objection which has been stated, the few following observations seem necessary.

"No observations, according to my recollection, have been recorded, by which the force and velocity of the circulation in the unborn fœtus have been ascertained. As some of the most important offices which the circulation serves after birth are otherwise supplied in the fœtus, the same vigorous circulation would not appear in that situation to be necessary. The equal distribution of heat through the body, and the preservation of it in the same temperature in different climates, offices requiring the aid of the circulation after birth, are before it indebted for the requisite aid to that of the mother. The frequent return of the blood to the lungs for renovation, so indispensible after birth, is, for the same reason, unnecessary in the fœtus. The chief office which the motion of the blood serves before birth, would appear to be the conveyance of nourishment to the different parts of the frame; and, to that purpose, a languid circulation would seem to be better adapted than a motion of greater rapidity.

"On reference to the part of the preceding inquiry in which the causes of the expansion of the chambers of the heart are detailed, it will be found that the walls of the ventricles of the heart derive the property of dilating themselves after contraction, to a certain degree, from their natural structure. In consequence of the circular direction of the muscular fibres, it was there contended that the dilatation of the chambers was the necessary effect of the simple relaxation of these fibres. This inference from the structure of the heart appeared to be confirmed by observation on the hearts of cold blooded animals, newly abstracted from the body.

"But upon considering the situation of the contents of the chest, and of the diaphragm of the fœtus, another cause will in this case be found for the dilatation of the heart after contraction. The diaphragm of the fœtus is not pushed up into the chest, as is generally supposed, but is drawn or held up to occupy a part of the space which is afterwards filled by the dilated lungs. This, so different from that which it assumes after birth, may easily be supposed to be a forced position of the diaphragm. The elastic substance which unquestionably enters into its composition, must be at all times, to a certain extent, upon the stretch. The heart, in consequence of the resiliency of the diaphragm, and the situation into which it is forced in the fœtus, must be drawn downwards from the lungs. The diminution of pressure arising among the contents contents of the chest, from the tendency which the diaphragm has to separate from the lungs, will necessarily, upon the fibres of the heart becoming relaxed, and its boundaries dilatable, cause an influx of blood from the veins into the organ; and the dilatation of its chambers, in the same manner, though, perhaps, not with the same force, as that with which the diastole of the heart has been supposed to be effected after birth by the resiliency of the lungs."

Whatever objections may be made to the above, we shall forbear to notice them. Our wish is to preserve our usual impartiality by as fair a selection as possible. If we have omitted any passage which would have thrown greater light upon his theory, our pages are open for the author's remarks, and our minds for his instructions.

---

*A Chemical Chart or Table, exhibiting an Elementary View of Chemistry, intended for the Use of Students and young Practitioners in Physic; also to revive the Memory of more experienced Persons; adapted for hanging up in public and private Libraries.* Dedicated, by permission, to George Pearson, Esq. M.D. F.R.S. Senior Physician to St. George's Hospital, of the College of Physicians of London, &c. &c.; by Robert Crowe, Surgeon in the Royal Navy.

The above title so amply expresses the intention of the work, that we shall only offer a general detail of the manner in which it is arranged, and a few specimens of the mode of execution.

The table is divided into three sections. The first contains a definition and general history of chemistry, as far as relates to its principal objects, viz. air, earths, alkalies, acids, &c. The second a description of the simple substances, with their usual arrangement. The third a short history and description of the compound combustibles; also an explanation of the secondary compounds and hydrosulphurets.

Under the first, after a very general history, are arranged the different species of earths, alkalies, and metals.

"Section the First.—*Chemistry,* in a general point of view, referable to those changes which are perpetually taking place amongst the ultimate or constituent particles of matter, may be divided into three parts:—1st. A description of the component parts of bodies, called *simple substances*, from not yet being decomposed or satisfactorily shewn to be compounds. 2d. An account of the compound bodies produced by the union of simple substances. 3d. An account of the nature of the power which induces the particles of different bodies to combine, called affinity, which is, according to the experiments of Sir H. Davy, nothing more than electrical attraction produced by the different electrical states of different kinds of matter.

"*Air*, Atmospheric, composed of oxygen and nitrogen in the proportion of about 22 of the former and 78 of the latter; contains also water, carbonic acid, hydrogen, &c.; is an elastic expansile fluid, necessary for the support of animal and vegetable life—sp. g. [i. e. specific gravity] 1 to 1000.

"*Water*. Composed of hydrogen and oxygen, proportion 85 by weight of the former, to 15 of the latter; found in a state of solidity, liquid, vapour, and combined with other bodies; contains in its liquid state a variety of substances; each substance, according to their nature, altering its properties, discovery of which forms an important object of chemical analysis; two methods adopted, viz. precipitation and evaporation.

"*Earths*, nine in number, composed of a metallic base united with oxygen; some are insoluble in water, some little or no taste, no smell, fixed, incombustible, and incapable, when pure, of being fused; specific gravity not exceeding 4. 9.

"*Alkalies* have a pungent acrid taste; neutralize acids turn vegetable blue colours to green, render oils miscible in water, and, when mixed with inflammable bodies, the compounds absorb oxygen. Three kinds, namely potash, soda, and ammonia; until lately the two first generally supposed to be elementary bodies. Sir H. Davy has discovered them to be decomposable, consisting of a metallic base united with oxygen. Ammonia, compounded of nitrogen and hydrogen. Its gas liquified by $\frac{1}{400}$ water—seldom pure, usually combined with carbonic acid: in this state they are termed carbonates; when deprived of this, pure or caustic.

"*Acids*. Word acid at present denotes all bodies which, when applied to the tongue, excite the sensation called sour; change the blue colour of vegetables to red; combine with alkalies, earths, and metallic oxides, producing a class of bodies called binary salts:—consist of oxygen, united to one or more bases. Some of the combustible bases combine with two doses of oxygen, sulphurous acid containing the smallest dose of oxygen, sulphuric containing a maximum of oxygen; agreed by chemists to denominate the double salts from the acids which they contain. The alkali, earth, or metallic oxide, combined with that acid, being the base of the salt; thus sulphate of soda denotes a compound of sulphuric acid and soda. Some acids combine with two bases at once, and form also what are called triple salts, distinguished by subjoining the names of both the bases; thus the salt composed of tartaric acid, potash, and soda, is called tartrate of potash with soda. Some salts combine with an additional dose of their acid; others with an additional dose of their base: the first distinguished by prefixing the preposition super to the usual name, the second that of sub.; e. g. super-sulphate of potash, and sub-bonate of soda."

After

After this follows an enumeration of all the alkalies, with a short description of each; next of the earths. We shall offer as specimens the last of these and the first of the metals, as they follow in order.

" *Glucina,* first discovered by Vauquelin in the two minerals called beril and emerald, sp. g. 2. 976—taste sweet.

" *Gold,* the most ductile and malleable of all the metals, imported principally from Spanish South America; when pure, soft, yellow colour, sp. g. about 19. 361. of considerable lustre, destitute of smell or taste, not altered by exposure to air, combines with most of the metals and forms a variety of alloys, may be beaten out into leaves only the 280,000th part of an inch, only soluble in nitro muriatic or chloric acid, from which it may be separated by tin, affording a precipitate of a beautiful purple colour. Gold coin contains one 12th of alloy, of silver or copper. One lb. of standard gold is coined into 44 half guineas."

The account of metals includes all the latest discovered.

The acids follow, with all their compounds, extremely well arranged for perspicuity and brevity, but in a manner which we cannot explain without imitating the table.

The second section includes the divisions of simple substances, hitherto undecompounded, commonly, but without sufficient knowledge, called elements. These are, first, imponderable or unconfinable substances, viz. light, caloric, and electric matter; second, substances which make the basis of acids, and maintain combustion, as oxygen, chlorine, fluorine, and iodine; third, inflammable, and denominated simple combustibles, as hydrogen, charcoal, sulphur, and phosphorus; fourth, uninflammable and unmetalized basis of acids, called simple incombustibles, as nitrogen and baron or boracic acid deprived of oxygen by the ingenious discovery of Sir Humphry Davy; fifth division is confined to metals.

The third section contains the *compound combustibles*, usually composed of carbon, hydrogen, and oxygen. These are very numerous, comprehending almost the whole of the animal and vegetable kingdom. Those most important in chemistry and medicine are, however, with much propriety, comprised under alcohol-ethers, volatile or essential oils, fixed oils, and bitumens. The latter are divided into two classes, bituminous oils and bitumina: the first confined to naptha or petroleum, and mallua or sea-wax; the bitumens are more divided, their importance much increasing, as forming, with different proportions of other combinations, our pit-coal, the value of which is greatly enhanced by the application of its hydrogen to the gas lights.

The

The secondary compounds, from their complicated nature, can be only slightly touched upon.

"The term (says our author) secondary compounds means a combination of the primary compounds with each other;—may be arranged under the five following heads: first, combinations of earths with each other, and with metallic oxides; second, combinations of earths with alkalies, called glass; third, combinations of acids with alkalies, earths, and metallic oxides called salts; fourth, combinations of sulphuretted hydrogen with alkalies, earths, and metallic oxides called hydro-sulphurets; and, fifth, combinations of oils with alkalies, earths, and metallic oxides called soaps.

"Hydro-sulphurets, principally employed in chemical analysis, enable chemists to separate the metallic oxides from alkalies and earths. Their solutions precipitate almost all the metallic oxides from their solutions; distinguished by the colour, as silver, black; antimony, orange; arsenic, yellow; &c. &c."

Such is the nature of this table, the value of which we need not insist on. The study of chemistry is now become so complicated, that it might have appeared impracticable to condense its various objects. We can only say that Mr. Crowe has succeeded much better than we could have expected, that we have hung his table in our different studies, and doubt not shall often have occasion to refer to it. If, under these examinations, we should meet with errors or unsatisfactory information, we shall not fail to give him occasional hints for a future edition. At present, we can only regret several typographical errors, which are, however, too palpable and unimportant to mislead the reader.

---

